# Diverse Genetic Etiologies of Unilateral Polymicrogyria

**DOI:** 10.1002/ana.78169

**Published:** 2026-02-11

**Authors:** Abbe Lai, Jennifer E. Neil, Shyam K. Akula, Dina Amrom, Eva Andermann, Ann Bergin, Roberto Caraballo, Allen Y. Chen, John Gaitanis, Ganeshwaran H. Mochida, Jill M. Gotoff, Giorgi Kuchukhidze, Daphna Marom, Christelle Moufawad ElAchkar, Miriam Regev, Lance H. Rodan, Heather Olson, Bo Zhang, Annapurna Poduri, Diane D. Shao, Christopher A. Walsh, Edward Yang

**Affiliations:** ^1^ Division of Genetics and Genomics, and Howard Hughes Medical Institute Boston Children's Hospital Boston MA; ^2^ Neurogenetics Unit, Montreal Neurological Institute and Hospital, Department of Neurology and Neurosurgery McGill University Montreal Quebec Canada; ^3^ Neurogenetics Unit and Epilepsy Research Group, Montreal Neurological Institute and Hospital, Department of Neurology and Neurosurgery and Human Genetics McGill University Montreal Quebec Canada; ^4^ Division of Epilepsy and Clinical Neurophysiology, Department of Neurology Boston Children's Hospital Boston MA; ^5^ Department of Neurology Harvard Medical School Boston MA; ^6^ Department of Neurology Hospital de Pediatría “Prof. Dr. Juan P Garrahan” Buenos Aires Argentina; ^7^ Division of Rheumatology NYU Langone Health New York NY; ^8^ Department of Neurology and Pediatrics Hasbro Children's Hospital, The Warren Alpert Medical School of Brown University Providence RI; ^9^ Division of Genetics and Genomics and The Manton Center for Orphan Disease Research, Boston Children's Hospital, Department of Pediatrics Harvard Medical School Boston MA; ^10^ Pediatric Neurology Unit, Department of Neurology Massachusetts General Hospital Boston MA; ^11^ Department of Pediatrics and Neurology Geisinger Health System Danville PA; ^12^ Department of Neurology Christian Doppler University Hospital, member of the European Reference Network EpiCARE, Paracelsus Medical University of Salzburg Salzburg Austria; ^13^ Neuroscience Institute, Centre for Cognitive Neuroscience, Christian Doppler University Hospital Salzburg Austria; ^14^ The Genetics Institute and Genomics Center, Tel Aviv Sourasky Medical Center and School of Medicine, Faculty of Medical and Health Sciences Tel Aviv University Tel Aviv Israel; ^15^ The Danek Gertner Institute of Human Genetics, Sheba Medical Center, Tel Hasomer, Faculty of Medicine Tel Aviv University Tel Aviv Israel; ^16^ Department of Pediatrics and Neurology Harvard Medical School Boston MA; ^17^ National Institute of Neurological Disorders and Stroke Bethesda MD; ^18^ Broad Institute of MIT and Harvard Cambridge MA; ^19^ Department of Radiology Boston Children's Hospital, Harvard Medical School Boston MA

## Abstract

**Objective:**

Polymicrogyria (PMG) is one of the most common human malformations of cortical development and is often classified by its radiographic pattern of distribution. Unilateral polymicrogyria (uPMG) is a subtype of PMG affecting a portion or all of one cerebral hemisphere. As most PMGs occur bilaterally, there has been no specific investigation as to whether the genetic underpinnings of uPMG comprise a subset of or a distinct entity from bilateral PMG. In this study, our goal was to assess both the genetic etiology of uPMG and the value of diagnostic genetic testing in this setting.

**Methods:**

We conducted a retrospective analysis of clinical data from individuals with uPMG seen in the Brain Development and Genetics Clinic and/or research participants of the Walsh Laboratory at Boston Children's Hospital. The final study cohort included 35 individuals from 30 families who were diagnosed with uPMG on brain magnetic resonance imaging (MRI) and also underwent genetic testing.

**Results:**

A likely genetic cause was identified in 26.7% (8/30) of unrelated individuals with uPMG in this cohort and segregated within one family (10/35 total subjects). Recessive genetic causes included *ASPM*, *WDR62*, and *TMEM216*. Dominant causes included 22q deletion syndrome, *DYNC1H1*, *SCN3A*, and hereditary hemorrhagic telangiectasia (*HHT*) genes, *ACVRL1* and *ENG*. This is the first report of variants in *DYNC1H1*, *TMEM216*, and *ACVRL1* in association with uPMG.

**Interpretation:**

The genetic causes of bilateral PMG and uPMG can overlap, but some are unique to certain distributions of the malformation. Genetic explanations for uPMG are found at comparable rates to bilateral PMG, suggesting that germline testing for this unique presentation is warranted. ANN NEUROL 2026;99:1277–1286

## Introduction

Polymicrogyria (PMG) is one of the most common human malformations of cortical development. It is characterized by the presence of increased gyral frequency as well as distortion of sulcal anatomy, and it is often classified by its distribution in the brain.^1^ PMG can be localized to a portion of the brain, most commonly in the perisylvian regions, or be diffuse, affecting all or multiple areas of the brain.^2^ Several etiologies for the development of PMG have been established, including vascular insults, congenital infections, and genetic variants.^3^, ^4^ These etiologies can have other associated structural abnormalities of the brain. For example, additional cortical malformations such as schizencephaly or porencephalic clefts are seen with PMG secondary to destructive vascular etiologies,^5^ parenchymal calcifications or cysts are found with infectious etiologies such as congenital cytomegalovirus (CMV infection,^6^ and extracortical malformations such as abnormal basal ganglia cleavage or microcephaly are associated with some genetic forms of PMG.^7^

Unilateral polymicrogyria (uPMG) is a subtype of PMG affecting a portion or all of one cerebral hemisphere. Most known genetic etiologies of PMG result in a bilateral distribution (including asymmetric involvement) and are commonly thought to be unlikely contributors to uPMG.^7^, ^8^ Previous studies have asserted that uPMG may be primarily due to non‐genetic factors, such as a vascular insult, infection, or somatic genetic variation.^4^, ^6^, ^9^, ^10^ Several studies have supported the utility of genetic testing for PMG as a whole with a diagnostic yield estimated at 20 to 30%,^4^, ^11^ but the utility of genetic testing for uPMG has not been separately assessed. Therefore, this study aims to define the possible genetic etiologies for uPMG and to determine whether genetic testing for this group of disorders is warranted.

## Methods

This study was approved by the Institutional Review Board of Boston Children's Hospital for human subject research under protocol M08‐05‐0240 (Genetics of Neurological Conditions) that permits publishing de‐identified information obtained by retrospective record review with a waiver of informed consent, and protocol 05–05‐076R (Genetics of Epilepsy and Cognitive Disorders), in which all subjects provided written informed consent for collection and publication of de‐identified clinical information.

To investigate potential genetic causes of uPMG, a retrospective analysis was conducted of clinical information on patients with a unilateral malformation seen in the Brain Development and Genetics (BrDG) Clinic from 2013 to 2023 and research participants from the Walsh Laboratory enrolled from 1998 to 2023. The BrDG Clinic is a multi‐disciplinary specialty clinic at Boston Children's Hospital evaluating patients with various neurogenetic conditions and the Walsh Laboratory investigates malformations of cortical development with the goal of identifying and understanding their diverse genetic etiologies. Drawing from these 2 sources, which comprise over 600 probands with polymicrogyria, the relationships between genetic diagnoses and MCDs can be systematically evaluated, in this case for uPMG.

We identified a total of 82 individuals with possible uPMG from the BrDG clinic and Walsh Laboratory research study cohorts. We reviewed all available clinical records and magnetic resonance imaging (MRI) scans of each individual to ascertain cases that fit our inclusion criteria of radiographically confirmed uPMG, as defined by prior studies,^2^, ^12^ and a history of comprehensive prior genetic testing (either a brain malformation gene panel or exome sequencing). All brain MRI scans were reviewed by neuroradiologists for confirmation of radiographic inclusion criteria. Participants were excluded if no genetic testing was performed (19), if imaging was not available for review (14), if there was positive congenital CMV infection testing (1), or if the MRI showed no malformation (1), evidence of schizencephaly (1), heterotopias without PMG (2), or bilateral PMG (9). The reasons behind the lack of genetic testing for the 19 individuals included that testing was not pursued by the clinical team (9), there was insufficient sample and/or consent for genetic testing (8), or the research team had not yet conducted testing at the time of this review (2).

The final cohort consisted of 35 individuals from 30 families, including 19 male subjects, 15 female subjects, and 1 fetus of unknown biological sex. All genetic testing was performed on blood, saliva, or buccal samples. Most individuals had exome sequencing (31/35); 17 of these were trios, 7 were proband only, 2 were duos, and 1 was a multiplex family. Twenty‐eight of these exomes were performed by a prior research study through the Center for Mendelian Genomics (CMG) at the Broad Institute of Massachusetts Institute of Technology (MIT) and Harvard from 2019 to 2020. Briefly, this sequencing was performed using Illumina or Twist 150 bp paired‐end reads, covering over 85% of targeted regions at 20 × with an average coverage of 55 × and alignment was carried out using the BWA aligner against hg38. Variant calling was done using the Genome Analysis Toolkit (GATK) HaplotypeCaller version 3.5 and copy number variant (CNV) analysis was performed with exome sequencing data using the GATK‐gCNV pipeline. Three individuals (UNL101, PMG23601, and PH28401) had clinical exome sequencing between 2017 and 2023 through Clinical Laboratory Improvement Amendment (CLIA)‐certified laboratories and clinical reports were utilized for this analysis. Sequencing data was not available for independent review. The rest (4/35) had a custom multiple independent primer polymerase chain reaction (PCR) sequencing^13^ based gene panel that included 155 curated PMG‐associated genes from 2015 to 2016. More detailed descriptions of the research exome and panel sequencing methods are available in Akula et al.^9^

Data from the 28 Broad exome sequencing cases were reviewed using the seqr platform by at least 2 independent researchers in collaboration with the Broad Institute,^14^ whereas the data from the 4 individuals who underwent panel sequencing were analyzed by 2 independent researchers using an internal MySQL database. Plausible variants identified in the probands were interpreted according to updated American College of Medical Genetics and Genomics (ACMG) variant interpretation guideline best practices.^15^ Clinically reported variants were also reinterpreted in the same way. Variants were considered for pathogenicity if they met at least 2 ACMG pathogenicity criteria, and no benign criteria. Variants were filtered based on allele frequency thresholds, using a maximum allele frequency of 0.01 for recessive disorders and 0.0001 for dominant disorders, and had no homozygous occurrences in gnomAD version 4.1.0. Segregation within families was reviewed whenever possible, considering biallelic variants for recessive conditions and inherited or de novo heterozygous variants for dominant conditions. The predicted consequence of missense, synonymous, and intronic variants were evaluated using a combination of in silico functional prediction algorithms Combined Annotation Dependent Depletion (CADD; score > 20), predicted effect on splicing (if intronic) using Alamut Visual Plus, and amino acid and nucleotide conservation. Additionally, reported associations with disease in ClinVar and the published literature were reviewed. All variants identified through research‐based testing were orthogonally confirmed by Sanger sequencing or oligo array.

## Results

The clinical and radiological characteristics of the final cohort of 35 individuals are summarized in Table 1. All participants in the study had uPMG. Among them, right‐sided PMG was significantly more common, occurring in 27 individuals (77%), compared to left‐sided PMG, which was found in 8 individuals (23%). To determine whether the observed sidedness deviated significantly from the expected 50% frequency, we used a generalized estimating equation (GEE) with a logistic link and exchangeable correlation structure to account for intrafamilial correlation within the cohort. Statistical significance was then evaluated using a Wald test, which indicated that right‐sided PMG was statistically more frequent, with a *p* value of 0.003 (R‐scripts are provided in Supplementary Data S1). In addition to uPMG, other cortical abnormalities were identified in 11 (31%) individuals, including grey matter heterotopia (5), microcephaly (3), dysgyria (2), and ulegyria (1). Many individuals also had abnormalities in other brain structures, the most common of which were ventriculomegaly/white matter abnormalities (n = 28, 80%) and midbrain/brainstem abnormalities (n = 17, 49%). Basal ganglia/thalami (n = 10, 29%), hippocampus (n = 8, 23%), and cerebellum (n = 5, 14%) abnormalities were recurrent but less frequently noted. The anterior commissure, orbits, and myelination appeared normal for this cohort. Detailed radiographic information for each participant is provided in Supplementary Table S1. The most common shared clinical features in this cohort were seizures (n = 24, 69%), developmental delay (DD)/intellectual disability (ID; n = 24, 69%), and hemiplegia/hemiparesis (n = 19, 54%). Phenotypes affecting other organ systems were also noted in 17 (49%) individuals and implicated the ocular (8), cardiopulmonary (4), and gastrointestinal/ genitourinary (3) systems. Hearing loss was also found in 2 unrelated subjects. Detailed clinical information for each participant is provided in Supplementary Table S2.

**TABLE 1:** Summary of Clinical and Radiographic Findings in the uPMG Cohort

Gyral Abnormalities	Number of Cases Total, n = 35	Percentage of Cohort
Right‐sided PMG	27	77%
Left‐sided PMG	8	23%
Grey matter heterotopia	5	14%
Microcephaly	3	9%
Dysgyria	2	6%
Ulegyria	1	3%
Subcortical abnormalities		
WM abnormalities/ventriculomegaly	28	80%
Midbrain/brain stem abnormalities	17	49%
Basal ganglia/thalami abnormalities	10	29%
Hippocampus abnormalities	8	23%
Cerebellar abnormalities	5	14%
Clinical symptoms		
DD/ID	24	69%
Seizures, febrile and a‐febrile	24	69%
Movement/Gait abnormalities	19	54%
Dysmorphic features	8	23%
Visual/ocular abnormalities	8	23%
Cardiopulmonary abnormalities	4	11%
Gastrointestinal abnormalities	3	9%
Hearing loss	2	6%

DD/ID = developmental delay/intellectual disability; PMG = polymicrogyria; WM = white matter.

**FIGURE 1:**
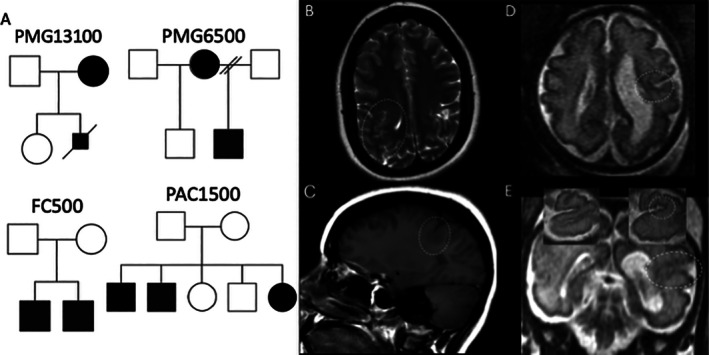
Pedigrees and selected MRI scans from multiplex uPMG families. (A) Pedigrees of multiplex families identified in this cohort. Axial T2 (B) and sagittal T1 (C) images of subject PMG13102 demonstrate right parietal polymicrogyria (*circled region*) and left parietal encephalomalacia. Single shot fast spin echo T2 weighted images from in utero images of PMG13101 in the axial plane (D), coronal plane (E), and right/left parasagittal planes (inset of D) demonstrate depressed volume in the left hemisphere, distortion of the left sylvian fissure, and increased gyral frequency suspicious for left sided polymicrogyria (*circled* region of interest). MRI = magnetic resonance imaging; uPMG = unilateral polymicrogyria.

**FIGURE 2:**
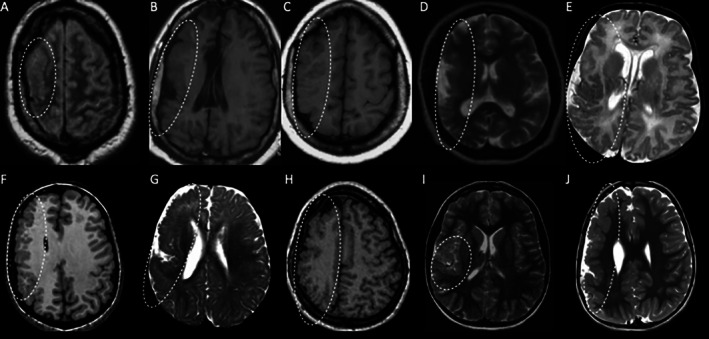
Montage of uPMG cases with suspected genetic diagnoses. Axial images are shown from PAC1501 (A), their brother PAC1502 (B), their sister PAC1503 (C), PS1901 (D), PMG8601 (E), BFP2901 (F), PMG12201 (G), PMG18401 (H), PMG2703 (I), and UNL101 (J). (B, C, F, H) Are T1‐weighted images and the remainder are T2‐weighted (A is T2* weighted). Circles denote areas of polymicrogyria. uPMG = unilateral polymicrogyria.

Four families in this analysis had multiple individuals with uPMG and their pedigrees are shown in Figure 1A. Two of the families were previously reported: FC500 with 2 affected sons and PMG6500 with an affected mother and son are “Family 1 Figure 2B” and “Family 3 Figure 2C,” respectively, in Chang et al. 2006.^16^ Two previously unreported multiplex families with uPMG are presented herein: PAC1500 has 3 affected siblings and PMG13100 has an affected mother and fetus. The familial cases exhibited marked intrafamilial radiographic similarities, except for family PMG13100 where the uPMG localized to different hemispheres (Fig 1B–E).

A likely germline genetic diagnosis for uPMG was found in 26.7% of families (8/30) comprising 10 affected individuals, 7 unrelated probands, and 3 siblings. This is a similar diagnostic yield to previous studies looking at populations with undifferentiated polymicrogyria,^2^, ^11^ The radiological and clinical information, variants, and ACMG classification criteria for all the genetic diagnoses are presented in Table 2. The genetic variants included 3 pathogenic variants, 2 likely pathogenic variants, and 5 variants of uncertain significance having met at least 2 ACMG pathogenicity criteria. The ClinVar ID for each variant is listed, if available.

**TABLE 2:** Radiological Features, Clinical Findings, and Genetic Variants in 10 Cases of uPMG

Proband	Cortical	White matter	Midbrain	Basal ganglia	Cerebellum	Microcephaly	Seizures	DD/ID	Neuro‐muscular	Gene/Region	Variant	Classification: ACMG criteria (ClinVar ID)
BFP2901	+	+	−	+	−	−	+	+	+	22q11.2	del (22) (q11.2)	P: N/A
PMG8601	+	+	+	−	+	N/A	N/A	+	+	*TMEM216*	NM_001173990.3 c.218G>T p.Arg73Leu	P: PS1, PS3 homozygous (197)
PS2703	+	−	−	−	−	−	+	−	N/A	*ENG*	NM_001114753.3 c.1470dup p.(Asp491Argfs*10)	P: PVS1, PS1, PM2 (862475)
PS10901	+	N/A	−	N/A	N/A	+	+	+	N/A	*ASPM*	NM_018136.5 c.7848_7849del p.(Ile2617Lysfs*19)	LP: PVS1, PM2 homozygous
UNL101	+	+	+	+	−	−	+	+	+	*ACVRL1*	NM_000020.3 c.266 G>T p.C89F	LP: PM2, PM5, PP1, PP3 (411299)
PAC1501	+	+	+	−	−	+	+	+	+	*WDR62*	NM_001083961.2 c.4561C>T p.Arg1521Trp c.758_760del p.Asn253del	VUS: PM2, PP1 (1307438) VUS: PM2, PM4, PP1 (3362467) in *trans*
PAC1502	+	+	+	−	−	+	+	+	+			
PAC1503	+	+	+	−	−	+	+	+	N/A			
PMG12201	+	+	+	+	−	−	−	+	+	*DYNC1H1*	NM_001376.5 c.6136C>T p.Arg2046Trp	VUS: PM2, PP2,PP3 (982939)
PMG18401	+	+	−	+	−	−	+	+	N/A	*SCN3A*	NM_006922.4 c.1862G>A p.Arg621His	VUS: PP2, PP3 (856037)

ACMG = American College of Medical Genetics and Genomics; DD/ID = developmental delay/intellectual disability; uPMG = unilateral polymicrogyria.

Both recessive and dominant genetic conditions are represented in this cohort. Biallelic diagnostic variants were identified in 5 individuals from 3 families. Three siblings (PAC1501, PAC1502, and PAC1503) have biallelic *WDR62* variants, PS10901 has a homozygous *ASPM* variant, and PMG8601 has a homozygous *TMEM216* variant. Heterozygous diagnostic variants were identified in the remaining 5 individuals from 5 families. BFP2901 has a 22q11 deletion, PMG12201 has a *DYNC1H1* variant, PMG18401 has a *SCN3A* variant, PS2703 has an *ENG* variant, and UNL101 has an *ACVRL1* variant. Several of the genes or loci identified herein are known to cause bilateral PMG but have rarely also been implicated in uPMG (eg, pathogenic variants in *ASPM*, *WDR62*, *ENG*, and *SCN3A*, as well as 22q11 deletion syndrome). The variants in *DYNC1H1*, *TMEM216*, and *ACVRL1* are associated with uPMG for the first time. Representative axial imaging highlighting the MRI findings in each of the solved cases is provided in Figure 2. The MRI scans for PS10901 have been previously published as Family 1 in Kuchukhidze et al. 2008, but the genetic diagnosis was not known at that time.^17^ Coronal and sagittal images for these cases are provided in Supplementary Figures S1 and S2.

## Discussion

We analyzed clinical, brain imaging, and genetic data from 35 individuals with uPMG across 30 families. A germline genetic cause was identified in 10 individuals from 8 families (26.7%), implicating 8 distinct genetic disorders. This yield for genetic testing in uPMG is similar to that previously described for undifferentiated PMG cohorts consisting primarily of bilateral PMG cases in which 18 to 30% have an identifiable genetic etiology.^11^, ^18^ Five of the 8 uPMG genetic diagnoses reported herein are also known causes of bilateral PMG (22q11.2 deletion, *SCN3A*, *ASPM*, *WDR62*, and *DYNC1H1*), one is unique to unilateral PMG presentations (*ENG*), one is associated with an unspecified PMG distribution (*TMEM216*), and one has not been previously associated with PMG before (*ACVRL1*).

Previously reported genetic causes of uPMG overlap of the genes and loci are identified in this study. The 22q11 deletion syndrome is one of the most common microdeletion syndromes and cortical malformations are a known but rare manifestation of this disorder. It was one of the first reported genetic causes of bilateral and unilateral polymicrogyria,^19^ and other studies have found that up to 9% of patients with PMG may have underlying 22q11 deletion syndrome.^2^ Additionally, a review of 24 individuals with 22q11 deletion found that more than half of the cases had radiographic findings on brain MRI, including 4 of 24 (16%) with cortical malformations.^20^ The prevalence of PMG in this condition may suggest that brain imaging is indicated for all individuals with 22q11 deletion, regardless of presenting symptoms.^14^, ^21^ *SCN3A* encodes a voltage‐gated sodium channel subunit that plays a critical role in the initiation and propagation of action potentials in neurons.^22^ Although most well‐known for causing a form of familial epilepsy, variants in *SCN3A* have also been associated with cortical malformations including uPMG.^22^, ^23^ The proband (PMG18401) with the *SCN3A* variant c.1862G>A p.(Arg621His) was previously reported as Family F in Smith et al.^22^ This variant is the only dominant variant in our cohort also present in gnomAD. However, we include this variant given its prevalence in gnomAD (0.00001239) is below our threshold for dominant disorders, in silico tools predict a damaging effect on protein function (CADD = 25.1), the arginine amino acid is highly conserved in *SCN3A* orthologs, and the missense constraint of *SCN3A* is high (z‐score = 6.37). *ASPM* and *WDR62* encode proteins important for proper centriole functioning and are 2 of the most common causes of autosomal recessive primary microcephaly.^24^ Cortical malformations rarely accompany primary microcephaly in these genes, but there have been single case reports of individuals with pathogenic variants in *ASPM* and *WDR62* having bilateral PMG,^25^, ^26^ as well as uPMG.^27^, ^28^

Novel causes of uPMG implicated in our cohort include *DYNC1H1* and *TMEM216*. *DYNC1H1* encodes the heavy chain of cytoplasmic dynein, a motor protein complex essential for intracellular transport, particularly in neurons.^29^ Pathogenic variants in *DYNC1H1* have been identified in individuals with bilateral PMG.^30^ However, we believe that this is the first report of a *DYNC1H1* variant associated with uPMG (PMG12201), and it was inherited from a father with a history of seizures and learning delays sufficient to require special education. Whereas brain imaging is not available for the father, the difference in severity of symptoms may be an indication of the variable expressivity of DYNC1H1‐related disorders, a phenomenon previously reported.^31^ *TMEM216* encodes a transmembrane protein which plays a crucial role in maintaining the structure and function of primary cilia.^32^ Variants in this gene are associated with a spectrum of ciliopathy syndromes including Joubert and Meckel syndromes. Brain MRI abnormalities include the “molar tooth sign,” an abnormal hindbrain, and, in the case of Meckel syndrome, encephaloceles.^32^ PMG associated with *TMEM216* has been previously reported in one individual,^33^ but the distribution was not described nor were brain images provided in the manuscript. Therefore, this is the first report of a radiographically confirmed case of uPMG associated with *TMEM216*.

*ENG* and *ACVRL1* are 2 of the genes which are known to cause hereditary hemorrhagic telangiectasia (HHT). HHT is a disorder characterized by abnormal blood vessel formation, leading to telangiectasias and arteriovenous malformations in various organs including the brain.^34^ Recently, a subset of individuals with pathogenic variants in *ENG* and uPMG have been reported,^34^, ^35^ We again demonstrate this association in our cohort but also extend it to another HHT gene, *ACVRL1*. This is the first report of an *ACVRL1* variant associated with PMG, which suggests a shared biological risk for the development of uPMG among HHT‐related genes.

Injury‐related PMG is often focal and can be associated with other malformations such as schizencephaly or manifestations of parenchymal destruction (eg, hemosiderin staining, gliosis, or parenchymal lysis). This association is exemplified by in utero hemorrhages in *COL4A1*‐related disorders,^5^, ^36^ Several of the genetic loci implicated in this report such as 22q11 deletion, *ENG*, and *ACVRL1*, are known to impact cardiovascular development, and the finding of uPMG without gliotic changes in these subjects suggests that uPMG may arise from cerebrovascular vulnerability during fetal life. This vulnerability could explain the occurrence of uPMG from a germline condition and merits further study.

A significant proportion of our uPMG cases were right‐sided (27), as compared to left‐sided (8). This right‐sided predominance persists, albeit to a lesser degree, even after inclusion of 19 subjects for whom genetic testing was not pursued: 10 of these 19 excluded cases were right‐sided such that 37 of 54 subjects with imaging (with or without genetic testing) had right‐sided uPMG. Statistical significance was again evaluated with the GEE and Wald testing, which showed right‐sided PMG was more frequent with a *p* value of 0.0134 (Supplementary Data S2). This degree of right‐sided predominance in our dataset has not been previously reported. Other studies have shown either similar numbers of right‐ and left‐sided uPMG^2^ or a slight left‐sided over‐representation,^4^ a discrepancy which may reflect differences in ascertainment criteria or enrichment for right‐sided uPMG in genetically solved cases. However, a right greater than left gradient of severity has been observed in some causes of bilateral polymicrogyria, such as those associated with *ATP1A3*,^37^ *AKT3*, *MTOR*,^38^ and chromosome abnormalities.^39^ Therefore, right‐sided over‐representation of uPMG may reflect developmental processes that are not yet fully understood (eg, asymmetry of cardiac or cerebral vasculature). Side to side differences in gyral maturation could also create windows of differential vulnerability to PMG. The cerebral hemispheres form asymmetrically during fetal development and the perisylvian area, a common area for PMG,^2^ is seen to appear earlier on the right hemisphere,^40^ although perinatal strokes are more commonly seen in the left hemisphere.^41^

Some candidate associations emerge from our data, suggesting potential clues to genetically identifiable causes of uPMG. All individuals with a genetic diagnosis had right‐sided uPMG and some degree of ID and/or DD. Co‐occurring microcephaly and PMG together was only ever observed with a genetic diagnosis (*ASPM* and *WDR62*), whereas grey matter heterotopia and PMG seen together were only observed in cases with negative genetic testing. Molar tooth sign and PMG were also only observed together in a genetically solved case. However, due to the limited number of individuals, the extent to which the association of these clinical features with genetically solvable uPMG can be generalized is unclear.

**FIGURE 3:**
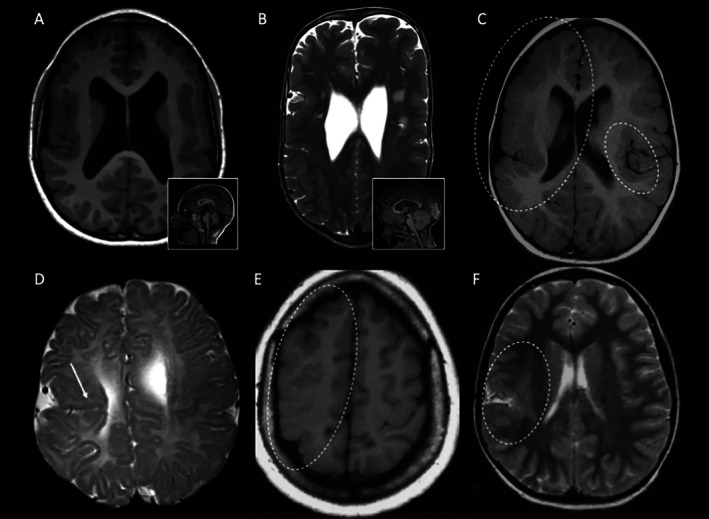
Spectrum of genetic and environmental manifestations of polymicrogyria. Axial MRIs from patients with congenital CMV (A) and *PIK3CA*‐related syndrome (B) demonstrate extensive bilateral PMG as expected for environmental or germline genetic alterations. Germline variants in other genes such as *TUBB2B* (C) can cause asymmetric bilateral malformations. Known vascular etiologies for PMG such as *COL4A1* (D) often have accompanying malformations as exemplified by the arrow in D indicating closed lip schizencephaly with adjacent polymicrogyria. *WDR62* (E), and *ENG* (F; higher axial slice from PMG2703, corresponding to the same individual shown in Fig 2I) demonstrate unilateral malformations. Inset images in A and B demonstrate sagittal profiles associated with microcephaly for CMV and macrocephaly for *PIK3CA*, respectively. Circles in C to F indicate areas of polymicrogyria. Panels A, C, and E are T1 weighted images whereas panels B, D, and F are T2 weighted images. CMV = cytomegalovirus; MRI = magnetic resonance imaging; PMG = polymicrogyria.

In summary, germline genetic etiologies explain a significant proportion of uPMG cases and uPMG cases cannot be assumed to have an environmental/non‐genetic explanation. More broadly, these results emphasize that PMG has myriad manifestations where environmental/injury‐related malformations could be bilateral and asymmetric/unilateral malformations could be genetic^42^, ^43^ (see Fig 3). Our study demonstrates that genetic etiologies of uPMG are heterogeneous and include genes seen in bilateral PMG as well as genes so far reported only in uPMG. Interestingly, right‐sided PMG was significantly more prevalent in this cohort, which may indicate hemisphere‐specific biological processes or developmental vulnerabilities yet to be elucidated. Given the comparable diagnostic yield to genetic testing for PMG as a whole, exome sequencing or a comprehensive gene panel warrants consideration for patients with uPMG to establish a molecular diagnosis, provide recurrence risk information, and inform further medical care.

## Limitations

A limitation of this study is the relatively small sample size of 35 cases, which may not fully capture the genetic heterogeneity underlying uPMG. Additionally, only 10 cases were found to have a genetic etiology, raising the possibility of missed somatic variants, novel gene‐disease associations, or new environmental/clinical factors not yet elucidated. Regarding potential somatic variants, the lack of access to affected brain tissue precluded somatic genetic testing in this cohort, and we cannot exclude low frequency somatic variants in blood or other tissues (eg, buccal mucosa) that might yield a genetic diagnosis with higher depth sequencing approaches. For the familial cases, only 1 of 4 pedigrees could be solved in our cohort and this limited number does not allow an accurate determination of yield rates for familial versus sporadic individual cases. A challenge in solving familial cases may be the limited sample availability from unaffected family members, which hampers assignment of pathogenicity by ACMG criteria and detection of causative variants due to insufficient segregation information. Last, functional and model system evaluation of the genes implicated in this cohort would be necessary to understand the precise mechanism contributing to uPMG, something that awaits further basic science investigation.

## Author Contributions

A.L., C.A.W., and E.Y., contributed to the conception and design of the study; A.L., J.E.N., D.A., E.A., A.B., R.C., J.C., J.M.G., G.K., D.M., M.R., G.H.M., C.M., S.K., A.C., L.H.R., H.O., B.Z., A.P. and D.D.S. contributed to the acquisition and analysis of data; A.L., J.E.N., and E.Y. contributed to drafting the text or preparing the figures.

## Potential Conflicts of Interest

The authors declare no conflicts of interest.

## Supporting information


**Supplementary Figure S1.** Coronal montage of unilateral polymicrogyria cases. Coronal images are shown from PAC1501 (A), their brother PAC1502 (B), their sister PAC1503 (C), PS1901 (D), PMG8601 (E), BFP2901 (F), PMG12201 (G), PMG18401 (H), PMG2703 (I), and UNL101 (J). (B, C, F, H) Are T1‐weighted images and the remainder are T2‐weighted (A is T2* weighted). Circles denote areas of polymicrogyria.

**Supplementary Figure S2.** Sagittal montage of unilateral polymicrogyria cases. Sagittal images are shown from PAC1501 (A), their brother PAC1502 (B), their sister PAC1503 (C), PS1901 unavailable (D), PMG8601 (E), BFP2901 (F), PMG12201 (G), PMG18401 (H), PMG2703 (I), and UNL101 (J). (B, C, F, H) Are T1‐weighted images and the remainder are T2‐weighted (A is T2* weighted). Circles denote areas of polymicrogyria.

**Supplementary Table S1.** Radiographic information extracted from the brain MRIs available for the 35 individuals included in this study. Empty cells indicate a lack of additional information. AP = anterior–posterior; b/l = bilateral; CSF = cerebral spinal fluid; d = days; GMH = grey matter heterotopia; L = left; m = months; N/A = not available; nl = normal; PMG = polymicrogyria; R = right; s/p = status post; VM = ventriculomegaly; WM = white matter; y = years.
**Supplementary Table S2.** Detailed phenotype descriptions of the 35 individuals included in this study. Empty cells indicate a lack of additional information. CMA = chromosomal microarray; CMV = cytomegalovirus; FISH = fluorescence in situ hybridization; FT = full term; GDD = global developmental delay; GERD = gastroesophageal reflux disease; ID = intellectual disability; KB = Kleihauer‐Betke; L = left; N/A = not available; nl = normal; PROM = premature rupture of membranes; R = right; SD = standard deviation; SVD = spontaneous vaginal delivery; VNS = vagus nerve stimulation; w = weeks; y = years.

**Supplementary Data S1.** R‐script for the estimating equation (GEE) and Wald test for the cohort of 35 that underwent genetic testing.

**Supplementary** **Data S2.** R‐script for the estimating equation (GEE) and Wald test for the larger cohort of 54 cases with imaging.

## Data Availability

The data that support the findings of this study are openly available in dbGaP accession # phs001272 and phs000492.v4.p3.
