# Supplementary figures and images for: Diverse Genetic Etiologies of Unilateral Polymicrogyria
Source: Ann Neurol. 2026 Feb 11;99(5):1277–86. doi: 10.1002/ana.78169 (PMC13033188; doi:10.1002/ana.78169)

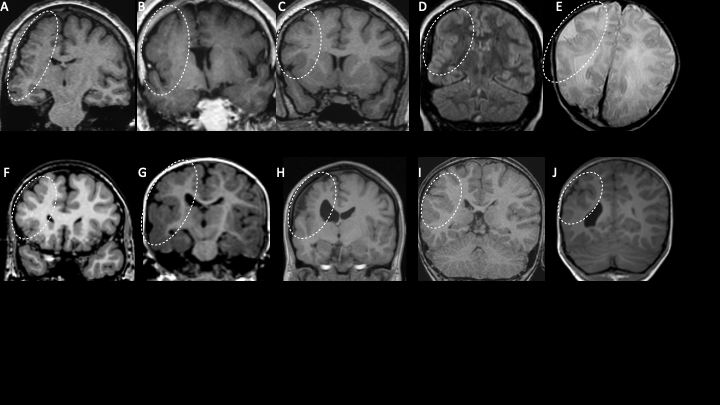

Supplement: Supplementary file 1 — Supplementary Figure S1. Coronal montage of unilateral polymicrogyria cases. Coronal images are shown from PAC1501 (A), their brother PAC1502 (B), their sister PAC1503 (C), PS1901 (D), PMG8601 (E), BFP2901 (F), PMG12201 (G), PMG18401 (H), PMG2703 (I), and UNL101 (J). (B, C, F, H) Are T1‐weighted images and the remainder are T2‐weighted (A is T2* weighted). Circles denote areas of polymicrogyria. [file ANA-99-1277-s003.tiff]

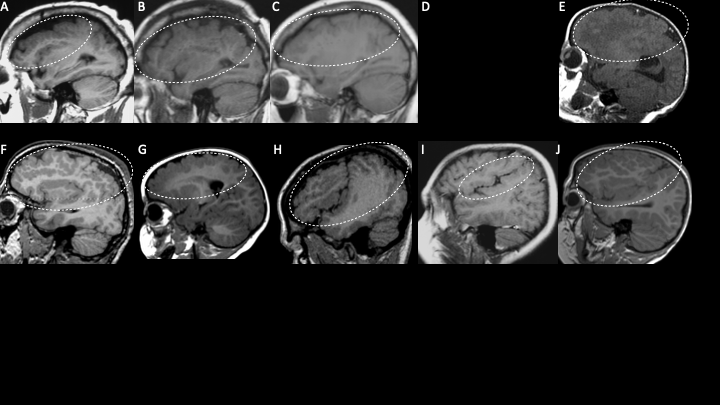

Supplement: Supplementary file 2 — Supplementary Figure S2. Sagittal montage of unilateral polymicrogyria cases. Sagittal images are shown from PAC1501 (A), their brother PAC1502 (B), their sister PAC1503 (C), PS1901 unavailable (D), PMG8601 (E), BFP2901 (F), PMG12201 (G), PMG18401 (H), PMG2703 (I), and UNL101 (J). (B, C, F, H) Are T1‐weighted images and the remainder are T2‐weighted (A is T2* weighted). Circles denote areas of polymicrogyria. [file ANA-99-1277-s005.tiff]
